# Molecular Analysis of *Escherichia coli* and Correlations Between Phylogroups and Sequence Types from Different Sources

**DOI:** 10.3390/microorganisms12122645

**Published:** 2024-12-20

**Authors:** João Gabriel Material Soncini, Vanessa Lumi Koga, Bruna Fuga, Zuleica Naomi Tano, Gerson Nakazato, Renata Katsuko Takayama Kobayashi, Nilton Lincopan, Eliana Carolina Vespero

**Affiliations:** 1 Department of Pathology, Clinical and Toxicological Analysis, Health Sciences Center, State University of Londrina, Londrina 86057-970, Paraná, Brazil; joaog.soncini@gmail.com (J.G.M.S.); vanessakoga68@hotmail.com (V.L.K.); naomi.tano@gmail.com (Z.N.T.); 2 Department of Cell Biology, Institute of Biological Sciences, University of Brasília, Brasília 70910-900, Goiás, Brazil; bruna.fuga@hotmail.com; 3 Department of Microbiology, Biological Science Center, State University of Londrina, Londrina 86057-970, Paraná, Brazil; gersonakazato@yahoo.com.br (G.N.); kobayashirkt@uel.br (R.K.T.K.); 4 Department of Microbiology, Biomedical Sciences Institute, University of São Paulo, São Paulo 05508-220, São Paulo, Brazil; lincopan@usp.br

**Keywords:** *Escherichia coli*, ESBL, virulence-associated genes, antimicrobial resistance, phylogroups, sequence type

## Abstract

*Escherichia coli* is a significant pathogen responsible for infections in both humans and livestock, possessing various virulence mechanisms and antimicrobial resistance that make it even more concerning. In this study, several internationally recognized clones of *E. coli* were identified, such as ST131, ST38, ST648, and ST354, from chicken meat, pork, and human infection samples. Notably, ST131, belonging to phylogroup B2, was the dominant sequence type (ST) in human samples, while ST38, belonging to phylogroup D, was the most prevalent in meat samples. Several antibiotic resistance genes were identified: the *gyrA* gene mutation was the most prevalent, and CTX-M-55 was the most common extended-spectrum beta-lactamases (ESBLs), with significant differences noted for CTX-M-2 and CTX-M-15. Virulence-associated genes (VAGs) such as *gad* and *iss* were frequently found, especially in human isolates. These findings highlight the complex epidemiology of antibiotic-resistant *E. coli* in community settings and the potential risks associated with commercial meat.

## 1. Introduction

*Escherichia coli* is one of the main bacterial pathogens associated with urinary tract infections (UTIs), accounting for a significant proportion of community-acquired infections [1]. Infections caused by *E. coli* strains producing extended-spectrum beta-lactamases (ESBLs) have become a major clinical challenge due to antibiotic resistance, resulting in limited treatment options and higher morbidity rates [2]. The spread of resistant strains, often associated with the inappropriate use of antimicrobials, raises concerns about public health and the effectiveness of antimicrobial therapies, especially in the context of increasing resistance in community settings [3].

In addition to infections in humans, *E. coli* is also frequently isolated from meats, especially chicken and pork, which are widely consumed in the human diet [4]. The use of antimicrobials in agriculture, aimed at promoting animal growth and preventing diseases, is associated with the development of bacterial resistance. The indiscriminate use of these medications in animal husbandry can lead to the selection of resistant strains, which, once introduced into the food chain, pose a significant risk to public health [3,5].

Antimicrobial resistance genes and virulence-associated genes (VAGs) can be easily transferred between different *E. coli* isolates. The CTX-M type is one of the largest groups of ESBLs and can be found on highly transferable plasmids [6]. Among *E. coli* uropathogenic (UPEC) isolates, CTX-M-15 is the most common; however, numerous CTX-M-producing *E. coli* strains have been identified as specific clones frequently associated with UTI cases in certain regions, and these clones are widely disseminated globally. Research indicates that CTX-M genotypes in food isolates may occasionally align with dominant human clones prevalent in the same area [7,8].

Additionally, VAGs play a crucial role in the pathogenicity of *E. coli*, enabling the bacterium to colonize, evade the host immune system, and cause disease. These genes encode factors such as adhesins, toxins, iron acquisition systems, and immune evasion mechanisms, which contribute to the ability of pathogenic *E. coli* strains to establish infections in diverse host environments. The distribution and prevalence of VAGs vary widely among different *E. coli* phylogroups and sequence types, reflecting their adaptation to specific niches [6,8,9].

*E. coli* ST131 is an example of a globally disseminated pandemic clone associated with CTX-M production, as well as other resistance mechanisms. Belonging to phylogroup B2, *E. coli* ST131 is also known for carrying numerous VAGs, making it highly pathogenic. Although it is strongly associated with urinary tract infections (UTIs), studies increasingly report its presence in veterinary, food, and environmental sources [6,9,10].

Other clones such as ST648, belonging to phylogroup F, have shown a significant increase in reports across human, veterinary, and environmental contexts in several countries, including Brazil, the United Kingdom, and India [11]. The dissemination of ST648 suggests a complex interconnection between human and animal reservoirs, making control of its spread challenging [12,13].

Given the significant impact of *E. coli* infections in Brazil on both human health and livestock, as well as the critical need to understand the spread of resistance and virulence genes, we conducted this epidemiological study from a One Health perspective. Our aim was to assess the transmission patterns of *E. coli* isolated from meat products and human patients in southern Brazil.

## 2. Materials and Methods

### 2.1. Study Population

This study includes 135 *E. coli* strains obtained from human urine samples (n = 59), chicken meat samples (n = 52), and pork samples (n = 24). These strains were previously selected and had their complete genome selected, as in a previously published study [4].

### 2.2. Bacterial Identification and Antimicrobial Susceptibility Test

The VITEK^®^ 2 AST 239 card and the VITEK^®^ 2 GN ID card (BioMérieux, Durham, NC, USA) were used for identification and antimicrobial susceptibility tests. Antimicrobial susceptibility was tested with 14 types of antibiotics: ampicillin, amoxicillin/clavulanate, ceftriaxone, cefepime, ertapenem, meropenem, nalidixic acid, ciprofloxacin, norfloxacin, gentamicin, amikacin, nitrofurantoin, trimethoprim/sulfamethoxazole, and piperacillin/tazobactam. In some cases, chromogenic agar medium (ChromID ESBL, BioMérieux, Marcy L’Etoile, France) was used to confirm for ESBL production.

The interpretation of the results was based on the CLSI 2020 (Clinical and Laboratory Standards Institute) criteria, and the *E. coli* ATCC^Ⓡ^ 25922 strain was used as a quality control [14].

### 2.3. ERIC-PCR

Enterobacterial Repetitive Intergenic Consensus (ERIC-PCR) was used to analyze the genetic similarity of the 1389 identified as ESBL-producing isolates. Genomic fingerprinting analysis was conducted using GelJ v.2.0 software and the Dice similarity method [15,16].

### 2.4. DNA Isolation and Whole-Genome Sequencing

For DNA extraction, the strains were cultured on Mueller–Hinton Agar and allowed to grow overnight at 37 °C. Subsequently, a single colony was inoculated into 2 mL of Luria–Bertani broth and incubated for 12 h at 37 °C. The resulting suspension was used for DNA extraction and purification using the DNA extraction kit from Invitrogen (Carlsbad, CA, USA). The extracted DNA was quantified using the Qubit dsDNA BR assay kit from Invitrogen (Carlsbad, CA, USA). Once quantified, the DNA was utilized to construct a paired-end library (150 bp) and subjected to sequencing on a NextSeq platform from Illumina (San Diego, CA, USA). All steps followed the instructions provided by the respective manufacturers.

### 2.5. Bioinformatic Analysis

Genome quality filtering and assemblies were performed using CLC Genomics Workbench version 7.0 (Aarhus, Denmark). Multilocus sequence type (MLST), resistome, and virulome were identified using MLST v2.0, ResFinder v3.1, VirulenceFinder v2.0, PlasmidFinder v2.1, FimTyper v1.0, and SerotypeFinder v.2.0, respectively [17,18,19,20,21,22].

### 2.6. Statistical Analysis

Categorical data were represented as frequencies and percentages. Fisher’s exact test or a chi-square test was applied as appropriate, considering an alpha significance level of 0.05. Data analysis was performed using Statistical Package for Social Sciences (SPSS—IBM Corp., New York, NY, USA), version 20, for Windows.

## 3. Results

**Figure 1:**
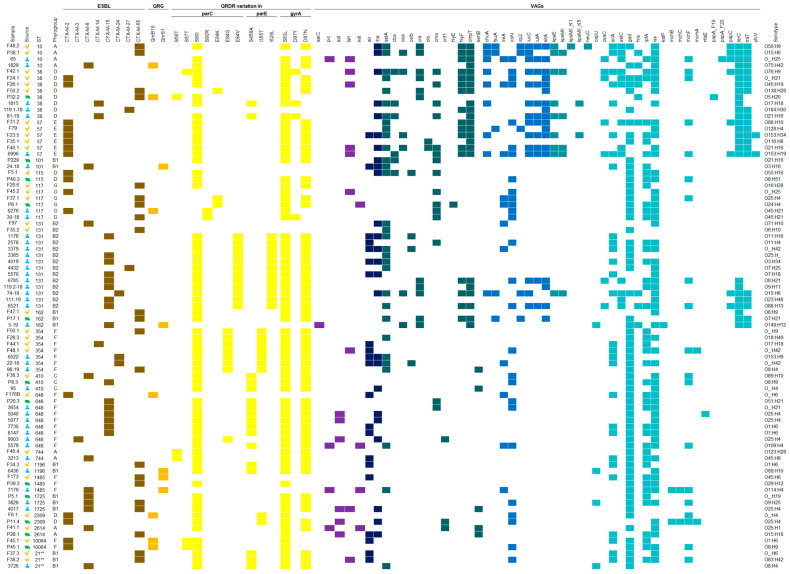
Heatmap. A visual scheme displaying the sample codes, sources, STs, phylogroups, ESBLs, quinolone resistance genes (QRGs), quinolone resistance-determining regions (QRDRs), VAGs, and serotypes. Color legend: In the source column, the yellow and green icons represent isolates derived from chicken and pork meat, respectively, while the blue icon represents those of human origin. The presence of ESBLs, QRGs, and QRDRs is represented by the colors brown, golden yellow and yellow, respectively. In the VAGs section, each color represents the classification of the gene: purple—effector delivery system; dark blue—adhesins; teal—exotoxin; blue—nutritional/metabolic factor; blue-green—capsule; light blue—other. The symbols ** represent *E. coli* scheme sequence type II (Institut Pasteur).

All data for each sample are compiled in Supplementary Materials (Complete Data Table), and a heatmap (Figure 1) was created with samples that shared the same ST in at least two of the studied sources.

### 3.1. Antibiotic Resistance Genes

Different resistance genes were found in the studied samples. The mutation in the *gyrA* gene was the most predominant resistance among the samples, being present in 88.5% (n = 46) of the chicken meat, 54.2% (n = 13) of the pork, and 79.7% (n = 47) of the human isolates. Two pork samples tested positive for the *mcr-1* gene (n = 8.3%). The most prevalent ESBL gene was *bla*_CTX-M-55_, positive in 42.3% (n = 22) of the chicken meat, 41.7% (n = 10) the pork, and 10.2% (n = 6) of the human samples.

Among the ESBLs genes that showed significant differences (Table 1) were *bla*_CTX-M-2_ (*p* = 0.002), *bla*_CTX-M-15_ (*p* = 0.001), and *bla*_CTX-55_ (*p* < 0.001).

### 3.2. Virulence-Associated Genes

The VAGs *gad* and *iss* were the most frequently found among the samples, being present in 63.5% (n = 33) and 78.8% (n = 41) of the chicken meat, 70.7% (n = 17) and 70.8% (n = 17) of the pork, and 96.6% (n = 57) and 72.9% (n = 43) of the human isolates, respectively.

Several prominent VAGs exhibited statistically significant differences across samples (Table 2). The exotoxin *hlyF* (*p* < 0.001) was detected exclusively in the chicken meat samples (40.4%) and pork samples (25.0%). The nutritional/metabolic factor *iutA* (*p* < 0.001), which was present in 38.5% of the chicken meat samples and 8.3% of the pork samples, *chuA* (*p* < 0.001), found in 26.9% of the chicken meat samples and 16.7% of the pork samples, and *fyuA* (*p* = 0.003), detected in 13.5% of the chicken meat samples and 16.7% of the pork samples, also demonstrated significant differences. Additionally, the genes classified as other factors, *gad* (*p* < 0.001), the most prevalent, and *papC* (*p* = 0.002), found in 15.4% of the chicken meat samples and 12.5% of the pork samples, highlighted their relevance.

Various *fimH* types were detected and are listed in Supplementary Materials (Table S1). The most prevalent were *fimH27*, present in six chicken meat samples (11.5%), one pork sample (4.2%), and twelve human samples (20.3%), and *fimH30*, found in one chicken meat sample (1.9%) and thirteen human samples (22.0%). Only *fimH30* showed a significant difference between the different sources (*p* < 0.0001).

### 3.3. Serotypes

Various serotypes were identified among the isolates, with the two most frequent ones also showing statistically significant differences, these being O25:H4 (*p* = 0.010), present in two (3.8%) chicken meat and nine (15.3%) human samples, and O1:H6 (*p* = 0.042), found in one (4.3%) pork and six (10.2%) human samples. All the O25:H4 strains belong to the ST131 clone, while the serotype O1:H6 is associated with ST648. All the results can be seen in Supplementary Materials (Table S2).

### 3.4. Sequence Types

A total of 55 different STs were identified, with the most prevalent being ST131 (n = 14), ST38 (n = 10), ST648 (n = 9), and ST354 (n = 7). The ST131 clone was predominantly found in human samples (n = 12) and in only two chicken meat samples. ST38 and ST648 were detected in all three different origins, with four and one from chicken meat, one and one from pork, and five and seven from humans, respectively. Lastly, ST354 was found in four chicken meat and three human samples.

### 3.5. Phylogroups

All phylogroups were identified in the studied samples, with B1 (n = 30) being the most prevalent, followed by A (n = 23), F (n = 23), B2 (n = 19), D (n = 19), E (n = 8), C (n = 6), and G (n = 6). Each phylogroup was detected in at least two different origins. Specifically, B1 (n = 14) and A (n = 12) were the most prevalent in human samples, while B2 (n = 17) was the most commonly found in chicken meat, and B1 (n = 7) was the most frequently diagnosed in pork samples. All isolates with the same ST belong to the same phylogroup.

## 4. Discussion

This study is a surveillance work that analyzed 135 *E. coli* isolates present in meats marketed for human consumption (chicken meat (n = 52) and pork (n = 24)) and in urine samples from community-acquired UTI patients (n = 59). The presence of internationally recognized clones among isolates from these different sources raises important questions about the spread of resistant and pathogenic *E. coli* lineages. The predominance of the ST131 clone, belonging to phylogroup B2, in human samples is well documented in the scientific literature, consistent with its strong association with clinical infections in both community and hospital settings [23,24]. In contrast, the ST38 (belonging to phylogroup D), ST648, and ST354 clones (belonging to phylogroup F) have been reported in both human infections and meat products and are considered emerging in the dissemination of antimicrobial resistance, particularly to quinolones and cephalosporins. Similar data can be found in the literature, with reports that ST38, phylogroup D, was found in 22% of the chicken samples and 14% of the UTI case samples, while ST648, phylogroup F, was identified in 10% of the pork samples and 8% of the human samples [4,25].

The dissemination of *E. coli* clones is closely associated with the spread of resistance genes. The CTX-M type represents one of the largest groups of ESBLs, and recent studies investigating the epidemiology of these enzymes in Brazil have highlighted CTX-M-2, CTX-M-8, CTX-M-9, and CTX-M-15 as predominant variants in this country [12]. Amongst all the samples in this study, nine types of *bla*_CTX-M_ genes were found; however, statistical differences were only found for the *bla*_CTX-M-2_ (*p* = 0.002), *bla*_CTX-M-15_ (*p* = 0.001), and *bla*_CTX-M-55_ (*p* < 0.001) genes. The *bla*_CTX-M-2_ and *bla*_CTX-M-55_ genes were detected in higher quantities in the chicken meat samples (n = 18 and n = 22, respectively), while the *bla*_CTX-M-15_ gene was more prevalent in the human urine samples (n = 14). These results are highly consistent with a study showing the proportion of isolates carrying the *bla*_CTX-M-2_ and *bla*_CTX-M-55_ genes as 78% and 45% in chicken samples and 15% and 5% in human samples, respectively, while the *bla*_CTX-M-15_ gene was more frequent in humans (45%) than in chickens (8%), suggesting that the transfer of these genes occurs more frequently within *E. coli* strains associated with either humans or chickens, and it may be less common among *E. coli* strains from different animal species. Increased interaction among members of the same animal species may contribute to the transfer of bacteria and bacterial genes among these populations [26].

The ESBL variants CTX-M-2 and CTX-M-55 are frequently found in *E. coli* isolated from chicken meat and pork, and their detection has been reported in countries such as the United States, the United Kingdom, France, and China. The beta-lactamase CTX-M-15 is endemic in various countries and is frequently associated with the epidemic clone *E. coli* ST131 (B2) which has worldwide distribution, primarily associated with human infections, as demonstrated by the data from the present study [27,28]. Although studies have been conducted on the chromosomal location of *bla*_CTX-M_ genes, plasmids are still considered the main vehicles for their rapid dissemination. Currently, it is reported that the *bla*_CTX-M_ genes are mainly located on plasmids *IncFII*, *IncI1*, *IncI2*, and *IncHI2* and often appear together with other antimicrobial resistance genes, as observed in our findings [29].

The frequency of coexistence of various resistance genes to different classes of antimicrobials highlights the complexity and adaptability of *E. coli* in response to the selective pressure exerted by antimicrobials. Furthermore, the coexistence of these genes in diverse sources, including humans, animals, and even the environment, suggests a broad and interconnected dissemination of antimicrobial resistance, known as One Health [26,30].

Although not statistically significant, the *mcr-1* gene was found only in the pork samples, which is consistent with published studies, where they reported a higher prevalence of the *mcr-1* gene in swine compared to other sources. Interestingly, it is known that fourteen distinct plasmid incompatibility groups are capable of carrying the *mcr-1* gene, with over 90% of globally identified plasmids belonging to the *IncX4*, *IncI2*, and *IncHI2* groups. In our study, the presence of the *IncX4* group was observed in all two *E. coli* isolates carrying the *mcr-1* gene, consistent with other studies indicating that the *IncX4* plasmid exhibits high transmissibility [31,32]. Additionally, the isolates belong to phylogroup B1, which is frequently reported in the literature as harboring the *mcr-1* gene [33,34].

Multiple VAGs were detected in our isolates and perform various mechanisms that assist in *E. coli* pathogenicity, enhancing its virulence and enabling the isolate to cause infections and damage to the host tissues. Among the VAGs that showed statistical differences between the groups, the ones that were prevalent in human urine samples were *sat* (*p* < 0.001), *cnf1* (*p* = 0.014), *senB* (*p* < 0.001), and *nfaE* (*p* = 0.006). Epidemiological data indicate a higher prevalence of these genes in UPEC when compared to other strains of *E. coli* [9]. Furthermore, in an interesting study using mouse models, it was demonstrated that *cnf1*-positive strains show increased colonization of the bladder, higher recovery rates in urine, and enhanced resistance to human neutrophil-mediated killing compared to *cnf1*-negative strains [35]. In this way, our results are consistent with those previously reported in the literature.

Most of the VAGs that showed significant differences were found in meat samples, like *iutA*, *kpsMII*, and *papC*. These genes were prevalent in the chicken meat isolates, consistent with results from other studies suggesting that APEC is the major carrier of resistance and virulence genes in chickens, responsible for losses in all types of poultry production systems. APEC is prevalent (9.52% to 36.73%) in all age groups of chickens, with broilers aged 4 to 6 weeks being the most susceptible to infection [36,37].

Recent research indicates that APEC is a potential zoonotic pathogen transmitted through food, as well as a source or reservoir of extra-intestinal infections in humans. Specifically, APEC shares genetic similarities with human ExPEC (UPEC), possessing virulence genes characteristic of UPEC, allowing it to cause UTIs [4,36,38]. This finding is further supported by our dates, as several VAGs are present in both our meat-derived isolates and in urine samples from patients with UTIs, with *astA* (*p* < 0.001), *cma* (*p* = 0.004), *capU* (*p* = 0.043), and *gad* (*p* < 0.001) showing statistically significant differences. Moreover, the identification of APEC-specific *ColV* (colicin V) plasmids in human UPEC isolates suggests a potential zoonotic transmission of APEC from avian sources to humans. Consequently, APEC represents a significant pathogen for both the poultry industry and public health [37,38].

Despite VAGs not being prevalent in pork compared to chicken meat isolates and human urine, it is known that these genes are also responsible for significant economic losses in the swine industry due to high morbidity, mortality, growth retardation, and increased therapy costs. Furthermore, they pose risks to human and poultry health due to horizontal gene transfer among *E. coli* from different sources [39,40].

With mechanisms of cell epithelial adhesion mediated by proteins, *fimH* becomes a crucial virulence factor for *E. coli* strains. Thus, the *fimH* gene is highly conserved and extremely common in *E. coli* isolates from different sources [41]. In our results, *fimH30* (*p* < 0.001) was the only one that showed statistically significant differences, being prevalent in human urine isolates. The *fimH30* variant is commonly associated with the pandemic *E. coli* ST131 lineage, a clone closely related to UTIs. In a study, *fimH* alleles of globally dominant UPEC ST131-B2 were evaluated, and it was observed that the *fimH30* variant has a greater ability to penetrate uroepithelial cells when compared to others, which explains the predominance of *fimH30* in *E. coli* derived from human urine [41,42].

Apart from adhesins, *E. coli* strains possess flagella, which contribute to bacterial mobility and serve as a significant antigenic component. The identification of serogroups is facilitated through the application of anti-O sera. Moreover, the antigenic diversity of flagella (H), combined with variability in O antigens, delineates the serotypes within the *E. coli* group. Among the prevalent serotypes linked to infections caused by UPEC strains are O1:H6 and O25:H4 [43,44]. These two serotypes showed statistically significant results in our study and were more prevalent in human urine isolates. An analysis of serotypes in 80 UPEC isolates in a study demonstrated that 51% belonged to classic serotypes such as O6:H1, O7:H4, O1:H6, O4:H5, O25:H4, O51:HNT, O34:HNM, and O8:H7, affirming our data [45].

One limitation of this study is that, although it used samples of meat sold for human consumption, *E. coli* isolates may have been introduced during processing, either by human handlers or by the instruments used to prepare the meat. Previous studies have shown that both equipment and workers’ hands can be significant sources of contamination [46,47]. Therefore, it is essential that further studies be conducted to provide a more comprehensive understanding of the epidemiology of *E. coli*.

In conclusion, *E. coli* is a complex microorganism characterized by various clones and serotypes, capable of acquiring diverse mobile genetic elements, which makes it a dangerous pathogen. The correlation between the presence of important clones in food products and their detection in clinical cases underscores the risk of transmission and highlights the urgent need for control strategies to mitigate the spread of resistant pathogens between humans and animals.

## Figures and Tables

**Table 1:** Prevalence and statistical analysis of antimicrobial resistance genes.

Antibiotic Resistance	Resistance Genes	Sources			*p*-Value
		Meats		Human	
		Chicken Meat n = 52 (%)	Pork n = 24 (%)	Urine n = 59 (%)	
**ESBL**	***bla*_CTX-M-1_**	1 (1.9)	0 (0.0)	0 (0.0)	1.000
	***bla*_CTX-M-2_**	18 (34.6)	3 (12.5)	4 (6.8)	0.002 *
	***bla*_CTX-M-3_**	0 (0.0)	0 (0.0)	1 (1.7)	0.437
	***bla*_CTX-M-8_**	6 (11.5)	3 (12.5)	9 (15.3)	0.615
	***bla*_CTX-M-14_**	1 (1.9)	0 (0.0)	6 (10.2)	0.043
	***bla*_CTX-M-15_**	0 (0.0)	3 (12.5)	14 (23.7)	0.001 *
	***bla*_CTX-M-24_**	0 (0.0)	0 (0.0)	2 (3.4)	0.189
	***bla*_CTX-M-27_**	0 (0.0)	0 (0.0)	3 (5.1)	0.081
	***bla*_CTX-M-55_**	22 (42.3)	10 (41.7)	6 (10.2)	<0.001 *
**β-lactam**	***bla*_CMY-2_**	8 (15.4)	2 (8.3)	3 (5.1)	0.147
	***bla*_SHV-12_**	2 (3.8)	0 (0.0)	0 (0.0)	0.504
	***bla*_OXA-1_**	0 (0.0)	1 (4.2)	6 (10.2)	0.043 *
	***bla*_TEM-1A_**	1 (1.9)	3 (12.5)	0 (0.0)	0.131
	***bla*_TEM-1B_**	22 (42.3)	14 (58.3)	28 (47.6)	1.000
	***bla*_TEM-141_**	9 (17.3)	6 (25.0)	0 (0.0)	<0.001 *
	***bla*_TEM-206_**	9 (17.3)	6 (25.0)	0 (0.0)	<0.001 *
	***bla*_TEM-209_**	0 (0.0)	1 (4.2)	0 (0.0)	1.000
	***bla*_TEM-214_**	9 (17.3)	6 (25.0)	0 (0.0)	0.005 *
**Sulphonamide**	***sul1***	25 (48.1)	7 (29.2)	36 (61.0)	0.037 *
	***sul2***	26 (50.0)	10 (41.7)	30 (50.8)	0.730
	***sul3***	6 (11.5)	1 (4.2)	1 (1.7)	0.137
**Tetracycline**	***tet(A)***	9 (17.3)	7 (29.2)	27 (45.8)	0.003 *
	***tet(B)***	10 (19.2)	10 (41.7)	17 (28.8)	0.846
	***tet(G)***	0 (0.0)	0 (0.0)	1 (1.7)	0.437
	***tet(M)***	0 (0.0)	0 (0.0)	1 (1.7)	0.437
**Fosfomycin**	***fosA***	15 (28.8)	8 (33.3)	3 (5.1)	<0.001 *
**Phenicols**	***catA1***	1 (1.9)	1 (4.2)	4 (6.8)	0.404
	**catB3**	0 (0.0)	0 (0.0)	7 (11.9)	0.021 *
	***cmlA1***	4 (7.7)	1 (4.2)	2 (3.4)	0.465
	***floR***	3 (5.8)	4 (16.7)	2 (3.4)	0.298
	***mdf(A)***	4 (7.7)	1 (4.2)	2 (3.4)	0.468
	***mph(A)***	1 (1.9)	3 (12.5)	26 (44.1)	<0.001 *
	***mph(B)***	0 (0.0)	1 (4.2)	0 (0.0)	1.000
**Trimethoprim**	***dfrA1***	4 (7.7)	3 (12.5)	10 (16.9)	0.314
	***dfrA5***	0 (0.0)	0 (0.0)	1 (1.7)	0.437
	***dfrA7***	2 (3.8)	0 (0.0)	0 (0.0)	0.504
	**dfrA8**	0 (0.0)	1 (4.2)	3 (5.1)	0.318
	***dfrA12***	3 (5.8)	0 (0.0)	5 (8.5)	0.296
	***dfrA14***	3 (5.8)	1 (4.2)	2 (3.4)	0.696
	***dfrA17***	6 (11.5)	3 (12.5)	27 (45.8)	<0.001 *
	***dfrA25***	0 (0.0)	0 (0.0)	1 (1.7)	0.437
	***erm(42)***	0 (0.0)	0 (0.0)	1 (1.7)	0.437
	***erm(B)***	0 (0.0)	0 (0.0)	4 (6.8)	0.034 *
**Quinolones**	***QnrB19***	6 (11.5)	3 (12.5)	4 (6.8)	0.246
	***QnrS1***	1 (1.9)	0 (0.0)	4 (6.8)	0.167
	***rmtB***	0 (0.0)	0 (0.0)	1 (1.7)	0.437
**Aminoglycosides**	***aac(3)-Iv***	0 (0.0)	1 (4.2)	0 (0.0)	1.000
	***aac(3)-VIa***	19 (36.5)	2 (8.3)	4 (6.8)	0.002 *
	***aac(3)-IIa***	0 (0.0)	0 (0.0)	3 (5.1)	0.081
	***aac(3)-IId***	3 (5.8)	1 (4.2)	8 (13.6)	0.128
	**aac(6′)Ib-cr**	0 (0.0)	1 (4.2)	8 (13.6)	0.010 *
	***aph(6)-Id***	0 (0.0)	0 (0.0)	0 (0.0)	0.131
	***aph(3′)-Ia***	4 (7.7)	3 (12.5)	1 (1.7)	0.137
	***aph(3″)-Ib***	4 (7.7)	6 (25.0)	0 (0.0)	0.003 *
	***aph(3′)-Ic***	1 (1.9)	1 (4.2)	2 (3.4)	1.000
	***aph(4)-Ia***	1 (1.9)	1 (4.2)	1 (1.7)	1.000
	***ant(2″)-Ia***	2 (3.8)	0 (0.0)	0 (0.0)	0.504
	***aph(6)-Id***	4 (7.7)	6 (25.0)	0 (0.0)	0.035 *
	***aacA4***	0 (0.0)	0 (0.0)	2 (3.4)	0.189
	***aadA1***	32 (61.5)	9 (37.5)	15 (25.4)	0.001 *
	***aadA2***	5 (9.6)	1 (4.2)	6 (10.2)	0.763
	***aadA2b***	1 (1.9)	0 (0.0)	0 (0.0)	1.000
	***aadA5***	5 (9.6)	3 (12.5)	25 (42.4)	<0.001 *
	***aadA12***	0 (0.0)	1 (4.2)	0 (0.0)	1.000
	***aadA24***	0 (0.0)	1 (4.2)	0 (0.0)	1.000
	***aadB***	4 (7.7)	0 (0.0)	3 (5.1)	1.000
	***strA***	12 (23.1)	9 (37.5)	25 (42.4)	0.099
	***strB***	12 (23.1)	9 (37.5)	25 (42.4)	0.099
**Fluroquinoles**	***parC*:p.S56T**	1 (1.9)	1 (4.2)	1 (1.7)	1.000
	***parC*:p.S57T**	2 (3.8)	1 (4.2)	0 (0.0)	0.256
	***parC*:p.S80I**	37 (71.2)	16 (66.7)	43 (72.9)	0.707
	***parC*_S80R**	0 (0.0)	0 (0.0)	2 (3.4)	0.189
	***parC*_E84A**	0 (0.0)	0 (0.0)	1 (1.7)	0.437
	***parC*:p.E84K**	2 (3.8)	2 (8.3)	0 (0.0)	0.131
	***parC*:p.E84G**	4 (7.7)	0 (0.0)	4 (6.8)	0.729
	***parC*_E84V**	0 (0.0)	0 (0.0)	12 (20.3)	<0.001 *
	***parE*_L416F**	0 (0.0)	0 (0.0)	4 (6.8)	0.034 *
	***parE*:p.S458A**	4 (7.7)	7 (29.2)	14 (23.7)	0.187
	***parE*:p.I355T**	6 (11.5)	1 (4.2)	3 (5.1)	0.512
	***parE*_I529L**	0 (0.0)	0 (0.0)	12 (20.3)	<0.001 *
	***gyrA*:p.S83L**	46 (88.5)	13 (54.2)	47 (79.7)	0.348
	***gyrA*:p.D87Y**	1 (1.9)	8 (33.3)	3 (5.1)	1.000
	***gyrA*:pD87N**	33 (63.5)	13 (54.2)	41 (69.5)	0.365
**Colistin**	***mcr-1***	0 (0.0)	3 (12.5)	0 (0.0)	0.256
**Peroxide**	***sitABCD***	19 (36.5)	7 (29.2)	0 (0.0)	<0.001 *
**Quaternary**	***qacE***	14 (26.2)	4 (16.7)	0 (0.0)	<0.001 *
**Aldehyde**	***formA***	3 (5.8)	3 (12.5)	0 (0.0)	0.035 *
**Lincosamide**	***Inu(A)***	1 (1.9)	2 (8.3)	0 (0.0)	1.000
	***cmIA1***	4 (7.7)	0 (0.0)	0 (0.0)	0.256

* Indicates significant difference.

**Table 2:** Prevalence and statistical analysis of virulence-associated genes.

Genes		Virulence-Associated Genes	Sources			*p*-Value
			Meats		Human	
			Chicken Meat, n = 52 (%)	Pork, n = 24 (%)	Urine, n = 59 (%)	
Adhesins	*afaD*	Adhesin Afa	1 (1.9)	0 (0.0)	0 (0.0)	0.563
	*air*	Enteroaggregative immunoglobulin repeat protein	10 (19.2)	5 (20.8)	18 (30.5)	0.149
	*iha*	Adherence protein	18 (34.6)	3 (12.5)	17 (28.8)	0.88
Effector delivery system	*aaiC*	Chromosomal gene	0 (0.0)	1 (4.2)	0 (0.0)	0.563
	*pic*	Serin protease autotransporter	2 (3.8)	0 (0.0)	3 (5.1)	0.653
	*sat*	Secreted autotransporter toxin	0 (0.0)	0 (0.0)	15 (25.4)	<0.001 *
	*tsh*	Temperature-sensitive hemagglutinin	13 (25.0)	2 (8.3)	5 (8.5)	0.068
	*vat*	Vacuolating autotransporter toxin	2 (3.8)	0 (0.0)	6 (10.2)	0.066
Exotoxin	*astA*	EAST-1 heat-stable toxin	23 (44.2)	10 (41.7)	6 (10.2)	<0.001 *
	*cba*	Colicin B	8 (15.4)	0 (0.0)	0 (0.0)	0.01 *
	*cea*	Colicin E1	6 (11.5)	4 (16.7)	0 (0.0)	0.003 *
	*celb*	Endonuclease colicin E2	3 (5.8)	1 (4.2)	0 (0.0)	0.131
	*cia*	Colicin Ia	9 (17.3)	6 (25.0)	0 (0.0)	<0.001 *
	*cib*	Colicin Ib	2 (3.8)	0 (0.0)	0 (0.0)	0.504
	*cma*	Colicin M activity	20 (38.5)	2 (8.3)	5 (8.5)	0.004 *
	*cnf1*	Cytotoxic necrotizing factor 1	0 (0.0)	0 (0.0)	5 (8.5)	0.014 *
	*hlyE*	Hemolysin E	1 (1.9)	1 (4.2)	0 (0.0)	0.504
	*hlyF*	Hemolysin F	21 (40.4)	6 (25.0)	0 (0.0)	<0.001 *
	*ompT*	Outer membrane protease	26 (50.0)	13 (54.2)	0 (0.0)	<0.001 *
	*senB*	Enterotoxin SenB/TieB	0 (0.0)	0 (0.0)	10 (16.9)	<0.001 *
Nutritional/ metabolic factor	*chuA*	Outer membrane hemin receptor	14 (26.9)	4 (16.7)	0 (0.0)	<0.001 *
	*fyuA*	Yersiniabactin siderophore receptor	7 (13.5)	3 (12.5)	0 (0.0)	0.003 *
	*ireA*	Iron-responsive element	8 (15.4)	4 (16.7)	4 (6.8)	0.178
	*iroN*	Salmochelin siderophore receptor	27 (51.9)	5 (20.8)	16 (27.1)	0.071
	*irp2*	Yersiniabactin	7 (13.5)	3 (12.5)	0 (0.0)	0.003 *
	*iucC*	Aerobactin synthetase	21 (40.4)	3 (12.5)	0 (0.0)	<0.001 *
	*iutA*	Aerobactin siderophore receptor	20 (38.5)	2 (8.3)	0 (0.0)	<0.001 *
	*sitA*	Iron/manganese transport	23 (44.2)	8 (33.3)	0 (0.0)	<0.001 *
Capsule	*kpsE*	Capsule polysaccharide export inner membrane protein	12 (23.1)	3 (12.5)	0 (0.0)	<0.001 *
	*kpsMII*	Group capsular II	7 (13.5)	2 (8.3)	0 (0.0)	0.005 *
	*kpsMII_K1*	Group capsular II	3 (5.8)	0 (0.0)	0 (0.0)	0.256
	*kpsMII_k5*	Group capsular II	2 (3.8)	1 (4.2)	0 (0.0)	0.256
	*neuC*	Polysialic acid capsule biosynthesis protein	3 (5.8)	0 (0.0)	0 (0.0)	0.256
Invasion	*ibeA*	Invasion of brain endothelium	1 (1.9)	0 (0.0)	0 (0.0)	0.376
Other	*aap*	Anti-aggregation protein	0 (0.0)	0 (0.0)	1 (1.7)	0.437
	*capU*	Hexosyltransferase homolog	0 (0.0)	1 (4.2)	6 (10.2)	0.043 *
	*ccl*	Cloacin	0 (0.0)	0 (0.0)	1 (1.7)	0.437
	cvaC	Microcin C	12 (23.1)	3 (12.5)	0 (0.0)	<0.001 *
	*eatA*	Enterotoxigenic *E. coli* (ETEC) autotransporter A	0 (0.0)	0 (0.0)	1 (1.7)	0.437
	*eilA*	Salmonella HilA homolog	18 (34.6)	9 (37.5)	20 (33.9)	0.858
	*estC*	Putative type I secretion outer membrane protein	17 (32.7)	4 (16.7)	0 (0.0)	<0.001 *
	*gad*	Glutamate decarboxylase	33 (63.5)	17 (70.7)	57 (96.6)	<0.001 *
	*hra*	Heat-resistant agglutinin	8 (15.4)	6 (25.0)	0 (0.0)	<0.001 *
	*IpfA*	Long polar fimbriae	23 (44.2)	15 (62.5)	27 (45.8)	0.625
	*iss*	Increased serum survival	41 (78.8)	17 (70.8)	43 (72.9)	0.648
	*katP*	Plasmid-encoded catalase peroxidase	0 (0.0)	1 (4.2)	0 (0.0)	0.563
	*mchB*	ABC transporter protein MchB	0 (0.0)	0 (0.0)	2 (3.4)	0.189
	*mchC*	ABC transporter protein MchC	0 (0.0)	0 (0.0)	3 (5.1)	0.081
	*mchF*	ABC transporter protein MchF	16 (30.8)	4 (16.7)	13 (22.0)	0.566
	*mcmA*	Microcin M part of colicin H	0 (0.0)	1 (4.2)	2 (3.4)	0.581
	*nfaE*	Nonfimbrial adhesin	0 (0.0)	0 (0.0)	6 (10.2)	0.006 *
	*papA_F19*	P fimbriae A	1 (1.9)	0 (0.0)	0 (0.0)	0.563
	*papA_F20*	P fimbriae A	1 (1.9)	1 (4.2)	0 (0.0)	0.504
	*papC*	P fimbriae C	8 (15.4)	3 (12.5)	0 (0.0)	0.002 *
	*terC*	Tellurium ion resistance protein	28 (53.8)	15 (62.5)	0 (0.0)	<0.001 *
	*traT*	Outer membrane lipoprotein	24 (46.2)	12 (50.0)	0 (0.0)	<0.001 *
	*yfcV*	Fimbrial protein	4 (7.7)	2 (8.3)	0 (0.0)	0.035 *

* Indicates significant difference.

## Data Availability

The data presented in this study are openly available in FigShare at https://doi.org/10.6084/m9.figshare.27853665.v4.

## Supplementary Materials

Additional Supplementary Materials can also be accessed through Figshare, including additional files related to this study, such as tables and FASTA files: https://doi.org/10.6084/m9.figshare.27853665.v4 (accessed on 9 December 2024).

## References

[1] Zhou Y., Zhou Z., Zheng L., Gong Z., Li Y., Jin Y., Huang Y., Chi M. (2023). Urinary Tract Infections Caused by Uropathogenic *Escherichia coli*: Mechanisms of Infection and Treatment Options. Int. J. Mol. Sci..

[2] Husna A., Rahman M.M., Badruzzaman A.T.M., Sikder M.H., Islam M.R., Rahman M.T., Alam J., Ashour H.M. (2023). Extended-Spectrum β-Lactamases (ESBL): Challenges and Opportunities. Biomedicines.

[3] Majumder M.A.A., Rahman S., Cohall D., Bharatha A., Singh K., Haque M., Hilaire M.G. (2020). Antimicrobial Stewardship: Fighting Antimicrobial Resistance and Protecting Global Public Health. Infect. Drug Resist..

[4] Soncini J.G.M., Cerdeira L., Sano E., Koga V.L., Tizura A.T., Tano Z.N., Nakazato G., Kobayashi R.K.T., Aires C.A.M., Lincopan N. (2022). Genomic insights of high-risk clones of ESBL-producing *Escherichia coli* isolated from community infections and commercial meat in southern Brazil. Sci. Rep..

[5] Manyi-Loh C., Mamphweli S., Meyer E., Okoh A. (2018). Antibiotic Use in Agriculture and Its Consequential Resistance in Environmental Sources: Potential Public Health Implications. Molecules.

[6] Daga A.P., Koga V.L., Soncini J.G.M., De Matos C.M., Perugini M.R.E., Pelisson M., Kobayashi R.K.T., Vespero E.C. (2019). *Escherichia coli* Bloodstream Infections in Patients at a University Hospital: Virulence Factors and Clinical Characteristics. Front. Cell. Infect. Microbiol..

[7] Giedraitiene A., Pereckaite L., Bredelyte-Gruodiene E., Virgailis M., Ciapiene I., Tatarunas V. (2022). CTX-M-Producing *Escherichia coli* Strains: Resistance to Temocillin, Fosfomycin, Nitrofurantoin and Biofilm Formation. Future Microbiol..

[8] Mujahid F., Rasool M.H., Shafiq M., Aslam B., Khurshid M. (2024). Emergence of Carbapenem-Resistant Uropathogenic *Escherichia coli* (ST405 and ST167) Strains Carrying blaCTX-M-15, blaNDM-5 and Diverse Virulence Factors in Hospitalized Patients. Pathogens.

[9] Terlizzi M.E., Gribaudo G., Maffei M.E. (2017). UroPathogenic *Escherichia coli* (UPEC) Infections: Virulence Factors, Bladder Responses, Antibiotic, and Non-antibiotic Antimicrobial Strategies. Front. Microbiol..

[10] Kudinha T., Kong F. (2022). Possible step-up in prevalence for *Escherichia coli* ST131 from fecal to clinical isolates: Inferred virulence potential comparative studies within phylogenetic group B2. J. Biomed. Sci..

[11] Kocsis B., Gulyás D., Szabó D. (2022). Emergence and Dissemination of Extraintestinal Pathogenic High-Risk International Clones of *Escherichia coli*. Life.

[12] Ewers C., Bethe A., Stamm I., Grobbel M., Kopp P.A., Guerra B., Stubbe M., Doi Y., Zong Z., Kola A. (2014). CTX-M-15-D-ST648 *Escherichia coli* from companion animals and horses: Another pandemic clone combining multiresistance and extraintestinal virulence?. J. Antimicrob. Chemother..

[13] Byarugaba D.K., Erima B., Wokorach G., Alafi S., Kibuuka H., Mworozi E., Musinguzi A.K., Kiyengo J., Najjuka F., Wabwire-Mangen F. (2023). Resistome and virulome of high-risk pandemic clones of multidrug-resistant extra-intestinal pathogenic *Escherichia coli* (ExPEC) isolated from tertiary healthcare settings in Uganda. PLoS ONE.

[14] (2020). Performance Standards for Antimicrobial Susceptibility Testing. 30th Informational Supplement.

[15] Versalovic J., Koeuth T., Lupski J.R. (1991). Distribution of repetitive DNA sequences in eubacteria and application to fingerprinting of bacterial genomes. Nucleic Acids Res..

[16] Heras J., Domínguez C., Mata E., Pascual V., Lozano C., Torres C., Zarazaga M. (2015). GelJ—A tool for analyzing DNA fingerprint gel images. BMC Bioinform..

[17] Larsen M.V., Cosentino S., Rasmussen S., Friis C., Hasman H., Marvig R.L., Jelsbak L., Sicheritz-Pontén T., Ussery D.W., Aarestrup F.M. (2012). Multilocus Sequence Typing of Total-Genome-Sequenced Bacteria. J. Clin. Microbiol..

[18] Bortolaia V., Kaas R.S., Ruppe E., Roberts M.C., Schwarz S., Cattoir V., Philippon A., Allesoe R.L., Rebelo A.R., Florensa A.F. (2020). ResFinder 4.0 for predictions of phenotypes from genotypes. J. Antimicrob. Chemother..

[19] Kleinheinz K.A., Joensen K.G., Larsen M.V. (2014). Applying the ResFinder and VirulenceFinder web-services for easy identification of acquired antibiotic resistance and *E. coli* virulence genes in bacteriophage and prophage nucleotide sequences. Bacteriophage.

[20] Carattoli A., Zankari E., García-Fernández A., Larsen M.V., Lund O., Villa L., Aarestrup F.M., Hasman H. (2014). In Silico Detection and Typing of Plasmids using PlasmidFinder and Plasmid Multilocus Sequence Typing. Antimicrob. Agents Chemother..

[21] Roer L., Tchesnokova V., Allesøe R., Muradova M., Chattopadhyay S., Ahrenfeldt J., Thomsen M.C.F., Lund O., Hansen F., Hammerum A.M. (2017). Development of a Web Tool for *Escherichia coli* Subtyping Based on *fimH* Alleles. Diekema DJ, organizador. J. Clin. Microbiol..

[22] Joensen K.G., Tetzschner A.M.M., Iguchi A., Aarestrup F.M., Scheutz F. (2015). Rapid and Easy In Silico Serotyping of *Escherichia coli* Isolates by Use of Whole-Genome Sequencing Data. J. Clin. Microbiol..

[23] Alves W.O., Scavuzzi A.M.L., Beltrão E.M.B., De Oliveira É.M., Vasconcelos C.R.S., Rezende A.M., Lopes A.C.S. (2022). Occurrence of blaNDM-7 and association with blaKPC-2, blaCTX-M15, aac, aph, mph(A), catB3 and virulence genes in a clinical isolate of *Klebsiella pneumoniae* with different plasmids in Brazil. Arch. Microbiol..

[24] Tchesnokova V., Larson L., Basova I., Sledneva Y., Choudhury D., Solyanik T., Heng J., Bonilla T.C., Pham S., Schartz E.M. (2023). Increase in the community circulation of ciprofloxacin-resistant *Escherichia coli* despite reduction in antibiotic prescriptions. Commun. Med..

[25] Edwards T., Heinz E., Van Aartsen J., Howard A., Roberts P., Corless C., Fraser A.J., Williams C.T., Bulgasim I., Cuevas L.E. (2022). Piperacillin/tazobactam-resistant, cephalosporin-susceptible *Escherichia coli* bloodstream infections are driven by multiple acquisition of resistance across diverse sequence types. Microb. Genom..

[26] Valenzuela X., Hedman H., Villagomez A., Cardenas P., Eisenberg J.N.S., Levy K., Zhang L., Trueba G. (2023). Distribution of blaCTX-M-gene variants in *E. coli* from different origins in Ecuador. Med. Microecol..

[27] Fernandes M.R., Sellera F.P., Cunha M.P.V., Lopes R., Cerdeira L., Lincopan N. (2020). Emergence of CTX-M-27-producing *Escherichia coli* of ST131 and clade C1-M27 in an impacted ecosystem with international maritime traffic in South America. J. Antimicrob. Chemother..

[28] Zhang J., Zheng B., Zhao L., Wei Z., Ji J., Li L., Xiao Y. (2014). Nationwide high prevalence of CTX-M and an increase of CTX-M-55 in *Escherichia coli* isolated from patients with community-onset infections in Chinese county hospitals. BMC Infect. Dis..

[29] Pan Y.S., Zong Z.Y., Yuan L., Du X.D., Huang H., Zhong X.H., Hu G.Z. (2016). Complete Sequence of pEC012, a Multidrug-Resistant IncI1 ST71 Plasmid Carrying blaCTX-M-65, rmtB, fosA3, floR, and oqxAB in an Avian *Escherichia coli* ST117 Strain. Front. Microbiol..

[30] Ferreira J.C., Filho R.A.C.P., Andrade L.N., Junior A.B., Darini A.L.C. (2016). Evaluation and characterization of plasmids carrying CTX-M genes in a non-clonal population of multidrug-resistant Enterobacteriaceae isolated from poultry in Brazil. Diagn. Microbiol. Infect. Dis..

[31] Clemente L., Manageiro V., Correia I., Amaro A., Albuquerque T., Themudo P., Ferreira E., Caniça M. (2019). Revealing mcr-1-positive ESBL-producing *Escherichia coli* strains among Enterobacteriaceae from food-producing animals (bovine, swine and poultry) and meat (bovine and swine), Portugal, 2010–2015. Int. J. Food Microbiol..

[32] Madni W.A., Mohsin M., Nawaz Z., Muzammil S., Zahoor M.A., Asif R. (2024). Molecular mechanism of antimicrobial co-resistance Colistin (mcr-1) and ESBLs genes among *Escherichia coli* isolates from commercial chickens in Pakistan. Braz. J. Biol..

[33] Shen C., Feng S., Chen H., Dai M., Paterson D.L., Zheng X., Wu X., Zhong L.L., Liu Y., Xia Y. (2018). Transmission of *mcr-1* -Producing Multidrug-resistant Enterobacteriaceae in Public Transportation in Guangzhou, China. Clin. Infect. Dis..

[34] Torres R.T., Cunha M.V., Araujo D., Ferreira H., Fonseca C., Palmeira J.D. (2021). Emergence of colistin resistance genes (mcr-1) in *Escherichia coli* among widely distributed wild ungulates. Environ. Pollut..

[35] Whelan S., Lucey B., Finn K. (2023). Uropathogenic *Escherichia coli* (UPEC)-Associated Urinary Tract Infections: The Molecular Basis for Challenges to Effective Treatment. Microorganisms.

[36] Subedi M., Luitel H., Devkota B., Bhattarai R.K., Phuyal S., Panthi P., Shrestha A., Chaudhary D.K. (2018). Antibiotic resistance pattern and virulence genes content in avian pathogenic *Escherichia coli* (APEC) from broiler chickens in Chitwan, Nepal. BMC Vet. Res..

[37] Rezatofighi S.E., Najafifar A., Badouei M.A., Peighambari S.M., Soltani M. (2021). An Integrated Perspective on Virulence-Associated Genes (VAGs), Antimicrobial Resistance (AMR), and Phylogenetic Clusters of Pathogenic and Non-pathogenic Avian *Escherichia coli*. Front. Vet. Sci..

[38] Hu J., Afayibo D.J.A., Zhang B., Zhu H., Yao L., Guo W., Wang X., Wang Z., Wang D., Peng H. (2022). Characteristics, pathogenic mechanism, zoonotic potential, drug resistance, and prevention of avian pathogenic *Escherichia coli* (APEC). Front. Microbiol..

[39] Castro J., Barros M.M., Araújo D., Campos A.M., Oliveira R., Silva S., Almeida C. (2022). Swine enteric colibacillosis: Current treatment avenues and future directions. Front. Vet. Sci..

[40] Rhouma M., Fairbrother J.M., Beaudry F., Letellier A. (2017). Post weaning diarrhea in pigs: Risk factors and non-colistin-based control strategies. Acta Vet. Scand..

[41] Nasi G.I., Georgakopoulou K.I., Theodoropoulou M.K., Papandreou N.C., Chrysina E.D., Tsiolaki P.L., Iconomidou V.A. (2023). Bacterial Lectin FimH and Its Aggregation Hot-Spots: An Alternative Strategy against Uropathogenic *Escherichia coli*. Pharmaceutics.

[42] Liu C.M., Stegger M., Aziz M., Johnson T.J., Waits K., Nordstrom L., Gauld L., Weaver B., Rolland D., Statham S. (2018). *Escherichia coli* ST131-H22 as a Foodborne Uropathogen. mBio.

[43] Delannoy S., Beutin L., Mariani-Kurkdjian P., Fleiss A., Bonacorsi S., Fach P. (2017). The *Escherichia coli* Serogroup O1 and O2 Lipopolysaccharides Are Encoded by Multiple O-antigen Gene Clusters. Front. Cell. Infect. Microbiol..

[44] Flores-Oropeza M.A., Ochoa S.A., Cruz-Córdova A., Chavez-Tepecano R., Martínez-Peñafiel E., Rembao-Bojórquez D., Zavala-Vega S., Hernández-Castro R., Flores-Encarnacion M., Arellano-Galindo J. (2024). Comparative genomic analysis of uropathogenic *Escherichia coli* strains from women with recurrent urinary tract infection. Front. Microbiol..

[45] Hernández-Chiñas U., Ahumada-Cota R.E., Navarro-Ocaña A., Chávez-Berrocal M.E., Molina-López J., Rocha-Ramírez L.M., del Prado A.N., Eslava C.A. (2023). Phenotypic and genotypic characteristics of *Escherichia coli* strains isolated during a longitudinal follow-up study of chronic urinary tract infections. Front. Public Health.

[46] Lee K.Y., Lavelle K., Huang A., Atwill E.R., Pitesky M., Li X. (2023). Assessment of Prevalence and Diversity of Antimicrobial Resistant *Escherichia coli* from Retail Meats in Southern California. Antibiotics.

[47] Nehoya K.N., Hamatui N., Shilangale R.P., Onywera H., Kennedy J., Mwapagha L.M. (2020). Characterization of Shiga toxin-producing *Escherichia coli* in raw beef from informal and commercial abattoirs. PLoS ONE.

